# Uncommon Septic Arthritis of the Hip Joint in an Immunocompetent Adult Patient Due to *Bacillus pumilus* and *Paenibacillus barengoltzii* Managed with Long-Term Treatment with Linezolid: A Case Report and Short Literature Review

**DOI:** 10.3390/ph16121743

**Published:** 2023-12-18

**Authors:** Razvan Silviu Cismasiu, Rares-Mircea Birlutiu, Liliana Lucia Preoțescu

**Affiliations:** 1 Clinical Hospital of Orthopedics, Traumatology, and Osteoarticular TB Bucharest, B-dul Ferdinand 35-37, Sector 2, 021382 Bucharest, Romania; razvan.cismasiu@umfcd.ro; 2 Faculty of Medicine, University of Medicine and Pharmacy “Carol Davila”, 050474 Bucharest, Romania; liliana.preotescu@umfcd.ro; 3 National Institute for Infectious Diseases “Prof. Dr. Matei Bals”, 021105 Bucharest, Romania

**Keywords:** *Bacillus pumilus*, *Paenibacillus barengoltzii*, hip septic arthritis, case report, linezolid

## Abstract

The *Bacillus* and *Paenibacillus* genera are diverse soil-related bacterial pathogens. In this case report, we describe, to our knowledge, the first report of septic arthritis in a native hip joint in an immunocompetent adult patient caused by *Bacillus pumilus* and *Paenibacillus barengoltzii*. We describe the case of a 39-year-old Caucasian male patient who sought medical advice for chronic pain on the mobilization of the right hip, decreased range of motion, and physical asthenia. The patient underwent a surgical intervention (core decompression) for a right osteonecrosis of the femoral head, with a slightly favorable postoperative evolution after surgery for one month. Surgical treatment was planned on the basis of clinical and paraclinical investigations and the joint damage. The hip was explored using an anterior approach under spinal anesthesia and standard antibiotic prophylaxis. After resection of the femoral head, meticulous debridement of all inflammatory tissues was performed, and a preformed temporary spacer was inserted into the femoral canal. Bacteriological laboratory studies identified *Bacillus pumilus* and *Paenibacillus barengoltzii* via matrix-assisted laser desorption–ionization time-of-flight mass spectrometry analysis. The patient initially received nine days of empirical therapy with intravenous antibiotics (linezolid and meropenem). After the bacterial strains were identified, the patient received organism-specific antibiotic therapy with the same antibiotics and dose for eight days until discharge. After discharge, the patient was referred to another hospital, where he continued treatment with linezolid for seven weeks and, after that, four weeks of oral therapy with cotrimoxazole and rifampicin. During this period, no severe or potentially life-threatening adverse events were recorded during long-term treatment with linezolid or with the two oral antibiotics. In conclusion, our findings suggest that long-term treatment with linezolid may be a viable option for the management of bone and joint infections caused by *Bacillus pumilus* and *Paenibacillus barengoltzii*.

## 1. Introduction

Soil-borne bacterial and fungal pathogens are a serious threat to human health, particularly in primary healthcare settings. Among these, members of the *Bacillus* and *Paenibacillus* genera are Gram-positive, rod-shaped bacteria that are extensively distributed in the environment, including soil, air, water, and food [1,2,3]. Notably, these bacteria produce spores that possess remarkable resistance to heat, cold, and common disinfectants, thereby enabling their persistence on various surfaces for extended periods [3]. The genus *Paenibacillus* is an aerobic or facultatively anaerobic bacterium that is depicted as a rod-shaped Gram-positive or Gram-variable endospore-forming bacterium. Originally derived from a *Bacillus*, group 3 was proposed by Ash et al. [1] in 1993 based on comparative 16S RNA gene sequence analysis.

Only a few species belonging to these genera are considered medically relevant, such as *Bacillus anthracis*, the anthrax etiological agent, which is a notorious pathogen that can cause severe and often lethal disease in humans. *Bacillus cereus*, another member of this group, is commonly associated with food-borne intoxication and can cause local or systemic infections. When ingested, *B. cereus*-produced toxins can cause gastrointestinal (GI) and extra-gastrointestinal syndromes. GI syndromes, diarrheal illness, nausea, and vomiting without diarrhea. *B. cereus* has also been reported to be involved in eye, respiratory tract, and wound infections. Although many other species are generally considered contaminants in clinical cultures, recent reports suggest that they may have pathogenic potential in humans, both *Bacillus* spp. and *Paenibacillus* spp., causing local or systemic infections (endophthalmitis, cardiac device-related endocarditis, pacemaker lead infection, related bacteremia, infection secondary to esophageal perforation, sepsis in neonatal infants, urinary tract infections, and bacteremia in injection drug users) [4,5,6,7,8,9,10,11]. Identifying different species of the *Bacillus* genus using traditional methods can be challenging because of the similarities in morphological, biochemical, and genetic characteristics among closely related species. This issue is especially prevalent in the *B. cereus* sensu lato group, which includes species such as *Bacillus thuringiensis* and *Bacillus mycoides*, which are characterized by almost identical 16S rRNA gene sequences and show a high level of chromosomal synteny. However, matrix-assisted laser desorption–ionization time-of-flight mass spectrometry (MALDI-TOF MS) could be an effective diagnostic technique to overcome these challenges and identify these organisms. 16S rRNA gene sequence analysis is broadly considered the “gold standard” for bacterial identification [12,13,14].

Osteonecrosis of the femoral head (ONFH) occurs when the blood supply to the femoral head is damaged because of the terminal section of the vessels without anastomosis, leading to the death and subsequent repair of bone cells and bone marrow components. This, in turn, causes structural changes in the femoral head and joint collapse, resulting in pain and dysfunction. This condition is a typical refractory disease with a high disability rate, and most patients must undergo total hip arthroplasty. Core decompression (CD) is a treatment option that can reduce the pressure in the bone, open up the increased resistance zone that hinders the repair of osteonecrosis, stimulate the formation of new blood vessels around the decompression tunnel, enhance the replacement of new bone, and delay the progression of osteonecrosis. Regarding complications associated with CD surgeries, the results of a systematic review and meta-analysis of 2441 hips published by Hua et al. reported only five cases of infection (from 21 studies/1440 hips that recorded the type of complications) [15].

According to the European Bone and Joint Infection Society guidelines for the management of septic arthritis in native joints, for patients who are taking antibiotics at the time of synovial fluid aspiration, and in the context when difficult-to-culture pathogens are suspected, or in case of negative culture results despite a high suspicion of septic arthritis in native joints, molecular polymerase chain reaction (PCR) technology using synovial fluid is recommended [16]. The diagnosis of septic arthritis in native joints (SANJO) relies primarily on joint fluid aspiration, which is analyzed for synovial leukocyte count and bacterial identification. Empirical antibiotic treatment should be avoided, except in patients exhibiting signs of sepsis, until joint fluid sampling is performed to prevent false-negative culture results. Arthroscopic lavage (with synovectomy, depending on the clinical stage) is advisable for SANJO, particularly in larger joints. At the same time, open revision could be considered in cases of synovial membrane adhesion, cartilage, or bone damage. The selection of empirical antibiotic treatment should consider the most probable pathogens and be targeted based on results from the microbiology laboratory [16]. In terms of isolated pathogens from samples from patients with septic arthritis, *Methicillin-susceptible S. aureus* (MSSA) is the most common pathogen. It has been reported in approximately 45–65% of cases, followed by *Streptococcus* spp. (15%). *Methicillin-resistant S. aureus* (MRSA) rates have been reported to be as high as 5%. However, it is the most common pathogen in some regions, such as the US. Regarding Gram-negative bacteria (GNB), this type of pathogens is less frequently involved (15–17%), and the most commonly isolated are *P. aeruginosa* and *E. coli* [16].

Linezolid is an antibacterial agent that belongs to the oxazolidinone class and has a mechanism of action that inhibits the initiation of bacterial protein synthesis. It has an extensive spectrum of activity against Gram-positive bacteria, including methicillin-resistant staphylococci, penicillin-resistant pneumococci, vancomycin-resistant *Enterococcus faecalis,* and *E. faecium* strains. Linezolid is generally well-tolerated, with gastrointestinal impairments being the most frequently occurring adverse event. It is essential to mention that severe adverse reactions to linezolid may occur, including myelosuppression, peripheral and optic neuropathy, lactic acidosis, and serotonin syndrome, adverse reactions that require immediate withdrawal of the medication [17].

In summary, the *Bacillus* and *Paenibacillus* genera are a diverse group of soil-related bacterial pathogens with varying pathogenic potentials, and their ability to persist on environmental surfaces underscores the importance of appropriate infection control measures in clinical settings. No case reports of human infections caused by *Paenibacillus barengoltzii* have been published in our literature search. Regarding *Bacillus pumilus*, there is one case report from the orthopedics field of cervical spondylodiscitis in an immunocompetent patient [18].

In this case report, we describe, to our knowledge, the first report of septic arthritis of the hip joint in an immunocompetent adult patient due to *Bacillus pumilus* and *Paenibacillus barengoltzii*.

## 2. Case Report

We describe the case of a 39-year-old Caucasian male patient who lived in a rural area in Romania and sought medical advice for chronic pain (approximately 16 months) mobilization of the right hip, decreased range of motion of the right hip, and physical asthenia. From his past medical history, the patient was a heavy smoker with a 25 pack-year cigarette smoking history, and he is also known to have essential high blood pressure for which he is under treatment with an angiotensin-converting enzyme inhibitor, mixed dyslipidemia, and non-specific low back pain. In May 2022, in a different clinic, the patient underwent a core decompression intervention for right osteonecrosis of the femoral head, with a slightly favorable postoperative evolution for one month. Prior to admission into our hospital, the patient was examined in an outpatient clinic by an infectious disease specialist. At that time, from the laboratory examinations that were performed, a significant biological inflammatory syndrome was highlighted (C-reactive protein 73.8 mg/L—reference value 0–3 mg/L; erythrocyte sedimentation rate 68 mm/h—reference value 0–15 mm/h; fibrinogen 790 mg/dL ref. val. 200–393 mg/dL) and that the patient was referred to our hospital. Prior to his admission, magnetic resonance imaging (MRI) of the lumbar spine was performed and revealed no signs of spondylodiscitis.

On physical examination, the following changes were noticed at the time of admission: severely restricted movements of the right hip, and the joint exhibited slight warmth upon palpation. However, no evident presence of any visible swelling is observed. The patient was using for support and walking two crutches, and a right Harris Hip Score of 28.75 points was recorded.

In this context, an investigation protocol that included laboratory and complementary imaging investigations was initiated.

One set of blood specimens was drawn on the day of admission and was negative. Transthoracic echocardiography was also performed and revealed no signs of vegetation. Chest radiography revealed no significant abnormalities.

The main laboratory examinations performed on the day of admission are presented in Table 1 and highlight an important biological inflammatory syndrome.

**Figure 1:**
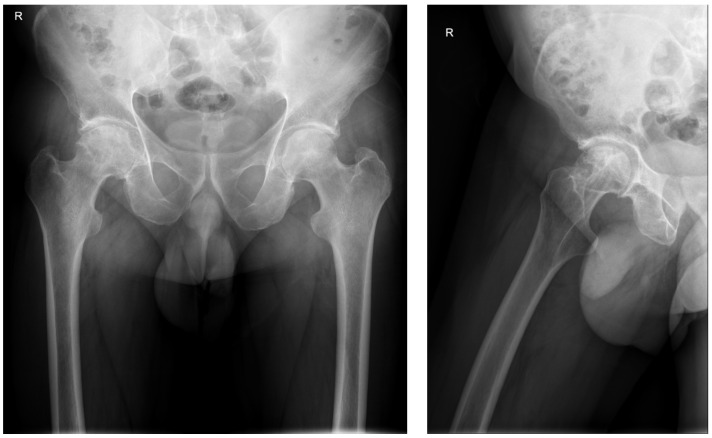
X-ray of the right hip on admission.

The initial radiography of the right hip joint showed a slight narrowing of the joint space and destruction of the femoral head (Figure 1).

**Figure 2:**
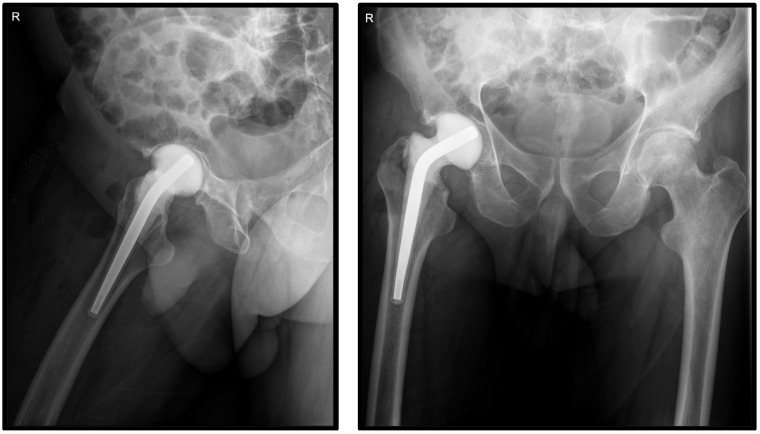
Postoperative X-ray assessment showing the antibiotic-loaded spacer.

Surgical treatment was planned on the basis of clinical and paraclinical performed investigations and the joint damage. The hip was explored using an anterior approach under spinal anesthesia and standard antibiotic prophylaxis, via an anterior approach. Prior to joint opening, synovial fluid was harvested for microbiological examination. After joint opening, five tissue and bone samples were harvested and sent to the laboratory for bacterial culture. After resection of the femoral head, accurate debridement of all inflammatory tissues was performed, and a preformed temporary spacer (Vancogenx^®^Space Hip, Tecres S.p.A., Sommacampagna, Verona, Italy) was implanted in the femoral canal (Figure 2). The vancomycin and gentamicin hip spacer (Vancogenx^®^) was loaded with a 1:1 concentration of antibiotics containing a combined total of 1.1 g to 3.2 g antibiotics.

In terms of bacteriological laboratory studies, the Gram stain was negative, and the acid-fast bacilli staining was negative. Five days after surgery, the growth of two strains of Gram-positive bacteria (from two samples) was observed without the possibility of identifying the bacterial species. The pure bacterial strains were sent to a reference laboratory for further analysis using MALDI-TOF MS analysis on a MALDI Biotyper Microflex LT mass spectrometer (Bruker Daltonik, Bremen, Germany). The isolates were subjected to duplicate testing. A bacterial colony was directly spotted on a MALDI plate and covered with 1 μL of saturated α-cyano-4-hydroxycinnamic acid. Subsequently, the plate was air-dried and introduced into the device according to the manufacturer’s instructions. The mass spectra that were obtained within 10 min were imported into integrated MALDI Biotyper software (version 3.0). The spectra were analyzed using standard pattern matching with default settings. A score of at least 2.00 indicated species-level identification and a score between 1.70 and 1.99 indicated genus-level identification. In contrast, any score below 1.70 indicated no significant similarity with any database entry. Four days after the strains were sent, *Bacillus pumilus* and *Paenibacillus barengoltzii* were isolated with the following minimum inhibitory concentrations (MICs) that were assessed according to the European Committee on Antimicrobial Susceptibility Testing (EUCAST) breakpoints (Table 2) for *Bacillus pumilus,* unfortunately, no clinical breakpoints have been established for *Paenibacillus* spp. Nevertheless, we assessed the MICs based on the EUCAST PK/PD (non-species related) clinical breakpoints and published MICs for *Bacillus spp*. In addition, both strains were tested for susceptibility to trimethoprim–sulfamethoxazole, and rifampicin, both susceptible strains, decisions made based on small, published data from the literature and case reports [19]. The MICs were assessed using the MICRONAUT system for antimicrobial susceptibility testing (AST) (MERLIN Diagnostika GmbH, Bornheim, Germany) from pure colonies that were isolated and suspended in sterile NaCl to obtain a 1.8–2.2 MacFarland concentration.

The patient initially received nine days of empirical intravenous antibiotic therapy (linezolid 600 mg PO/IV q12hr and meropenem 1 g q8hr). After the bacterial strains were identified, the patient received organism-specific antibiotic therapy with the same intravenous antibiotics and doses for eight days until discharge. Postoperative evolution was without any complications. Hip pain decreased dramatically. The patient was mobilized shortly after the surgery, and we encouraged him to undertake short weight-bearing walks using two crutches. Linezolid and meropenem were well tolerated without any adverse events, except for an increase in liver enzymes, for which the patient received hepatoprotective drugs (150 mg silymarin tablets, q12H for eight weeks).

The main laboratory examinations performed after the surgery and during hospitalization are presented in Table 3.

After discharge, the patient was referred to the “Matei Bals” National Institute of Infectious Diseases, where the patient continued his treatment with linezolid 600 mg PO/IV for seven weeks (for a total of 9 weeks of treatment with linezolid), followed by four weeks of oral therapy with cotrimoxazole 80 mg/400 mg, 2 tablets q8H, and rifampicin 300 mg, 2 tablets q12H, based on the small, published data from the literature. During this period, no mild/moderate/severe/potentially life-threatening adverse events were recorded during the long-term treatment with linezolid (total days of administration, 63 days) or with the two oral antibiotics at the last follow-up, and the liver enzymes were within normal range and also the proinflammatory markers. Owing to the favorable evolution of the patient, a total hip revision using the same surgical approach will be scheduled.

## 3. Discussion

### 3.1. Epidemiological and Clinical Considerations

Osteonecrosis of the femoral head is a debilitating condition that poses a significant challenge in orthopedic surgery. One potential solution is the implementation of core decompression. This surgical procedure facilitates the healing of osteonecrosis and promotes the development of new blood vessels and bone tissue around the decompressed area. However, it is essential to note that core decompression may carry the risk of septic arthritis. Thus, while core decompression may be a viable treatment option, it should be approached with caution, and the potential risks should be carefully weighed against the potential benefits [16]. Septic arthritis of the hip is a relatively uncommon but serious condition that can result in significant pain and disability for affected patients. The mortality rate associated with pathology is estimated to be approximately 10%. MSSA is the most frequently identified causative agent of septic arthritis of the hip. Unfortunately, culture-negative infections occur in an increased percentage of cases, ranging from 16.7% to 78.4% of cases [20]. Various factors influence the choice of treatment for hip infections in adult patients. However, selecting the most effective option relies on the type of infection, whether active or quiescent. Orthopedic surgeons have several surgical treatment options available, including lavage and debridement performed via an arthroscopic approach, resection arthroplasty (arthrotomy), and total hip replacement (THR) in one-step or two-step exchange when treating septic arthritis of the hip [20].

Opportunistic infections are rarely observed in healthy individuals, because a weakened immune system is a significant risk factor. Traditional biochemical and phenotypic techniques are commonly used in clinical microbiology laboratories to identify aerobic Gram-positive spore-bearing bacilli. Soil-related bacterial and fungal pathogens, both classic and emerging, can cause severe human diseases that are often encountered in primary care settings. *Bacillus* and *Paenibacillus* (previously known as *Bacillus* group 3) [1] are large, Gram-positive, rod-shaped bacteria that are widely distributed in the environment, including in soil, air, water, and food. These organisms form spores that are highly resistant to increased or reduced temperatures and disinfectants, facilitating their ability to survive under severe conditions on environmental surfaces for extended periods [2]. To date, only a few species belonging to these genera have been identified as medically involved. *Bacillus anthracis* is the causative agent of the acute and often fatal disease, anthrax. At the same time, *Bacillus cereus*, commonly known to cause food-borne intoxications, can also cause local and systemic infections [21]. Other species are typically regarded as clinically irrelevant and are generally considered contaminants in clinical cultures. However, recent studies have suggested that these organisms can cause local and systemic infections in humans. Identifying *Bacillus* species using conventional methods can be challenging not only because of similarities among related species with a common pattern of morphological characteristics but also in terms of biochemical and genetic ones [4,5,6,7,8,9,10,11].

*Bacillus pumilus* infections are not commonly reported in the literature, and case reports of human infections are scarce. The cases reported in the literature are usually associated with pediatric patients or conditions related to immunodepression. Additionally, some patients have shown a positive history of major surgery [5,22,23]. It has been reported that *Bacillus pumilus* infection can be linked to food poisoning, which occurs as a result of consuming meat dishes, eggs, baked goods, or tomato sauce. Symptoms of infection include fever, gastrointestinal symptoms, and bacteremia. In newborn and pediatric patients, sepsis caused by *Bacillus pumilus* is often associated with the placement of an intravenous catheter. In oncohematological patients undergoing chemotherapy, the infection can lead to bacteremia and, in severe cases, multiorgan failure and death [22,23,24]. In rare cases, localized infections can manifest as anthrax-like lesions, including skin ulceration, necrosis, erythema, and edema [25]. Diagnostic or therapeutic invasive procedures may also be considered potential risk factors, as seen in our case report, in which the patient underwent CD surgery prior to the onset of symptoms.

Bacteria belonging to the genus *Paenibacillus* are commonly found in various environments, including soils and plant roots. These rhizobacteria are essential for promoting plant growth and can be used in agriculture. Additionally, many species of *Paenibacillus* produce antimicrobial compounds with medicinal or pesticidal applications. The enzymes produced by these bacteria can also be used for bioremediation or to create valuable chemicals. However, some species of *Paenibacillus* are known to infect honeybees and other invertebrates. In contrast, others can cause occasional infections in humans [26].

In almost every published case, *Paenibacillus* infections were opportunistic and tended to be associated with immunocompromised patients. It has been associated with various diseases or syndromes such as chronic kidney disease, sickle cell disease, premature birth, Whipple’s disease, hydrocephalus, skin cancer, chronic interstitial nephropathy, and acute lymphoblastic leukemia. However, the relationship between the infection and the condition is not always clear, and it is possible that *Paenibacillus* simply occupies suitable niches opportunistically. Many *Paenibacillus* isolates recovered from humans come from elderly patients whose immune systems are generally weak [9,27,28,29,30,31].

### 3.2. Short Overview of Published Case Reports on Bacillus pumilus

Fusini et al. [18] report a C6–C7 intervertebral disc spondylodiscitis case in an immunocompetent patient. The authors performed an anterior open biopsy of the intervertebral disc to obtain adequate laboratory material for microbiological examination. Four microbiological specimens harvested during the surgery tested positive for *Bacillus pumilus*. All isolated strains were susceptible to the following antibiotics: ampicillin, amoxicillin, ciprofloxacin, clindamycin, erythromycin, imipenem, levofloxacin, teicoplanin, trimethoprim–sulfamethoxazole, and vancomycin. Based on these results, the authors initiated a six-week course of intravenous antibiotics (amoxicillin/clavulanic acid 2.2 g q8H plus ciprofloxacin 400 mg q12H). The antibiotic therapy was switched to oral therapy and was performed for another six weeks (amoxicillin/clavulanic acid 875/125 mg q8H in association with ciprofloxacin 500 mg q12H). Laboratory test results normalized after six weeks except for ESR, which returned to normal after eight weeks of treatment. The patient had a favorable outcome. This is in contrast to our strain of *Bacillus pumilus*, which was resistant to erythromycin and had intermediate susceptibility to ciprofloxacin and levofloxacin. Additionally, the strain in our case report was susceptible to carbapenems, vancomycin, and oxazolidinone. Differences were also observed in terms of the prescribed antibiotics. However, a longer treatment duration was required in both cases.

Other authors have also used ciprofloxacin in a case report of *Bacillus pumilus* bacteremia in a patient with food poisoning. In this case, the isolated strain was susceptible to commonly used antibiotics, except for cefepime and cefotaxime.

Borsa et al. [22] isolated a strain of *Bacillus pumilus* and found that it was resistant to commonly used antibiotics such as penicillin but susceptible to vancomycin, erythromycin, clindamycin, levofloxacin, and trimethoprim–sulfamethoxazole. The patient was initially treated with intravenous ceftriaxone (1000 mg q12H) in combination with metronidazole (500 mg q12H). Metronidazole treatment was discontinued after seven days, and ceftriaxone (1000 mg q12H) alone was continued for 14 days. The authors used a molecular technique to identify the pathogens, namely the MALDI-TOF-based VITEK^®^ MS system and 16S rRNA sequencing using the Illumina MiSeq^®^ platform. The isolated strains (two isolated strains) of *Bacillus pumilus* in the case of Kimouli et al. [8] from neonatal infants with severe sepsis were susceptible to penicillin, ampicillin, imipenem, vancomycin, erythromycin, levofloxacin, and trimethoprim–sulfamethoxazole, and only one strain was susceptible to clindamycin. In this case, vancomycin was administered (10 mg kg^−1^ body weight every 8 h for ten days) with a favorable outcome [8]. An overview of published case reports is summarized in Table 4.

### 3.3. Capabilities of Biofilm Formation

In terms of biofilm formation capabilities of the strains of *Bacillus pumilus* in Luria Bertani and EPS medium, all strains isolated by Calandroni et al. [3] were capable of forming biofilms. We can conclude that these strains can potentially cause biofilm-related infections in the field of bone and joint infections. It has also been reported in the literature in the area of bone and joint infections that opportunistic bacteria have a longer time to positivity of bacterial cultures of more than four days [32,33]; a more extended incubation period was also required in our case (5 days). Over the past few years, no changes have been observed in the local trends related to bone and joint infections, except for the increased presence of opportunistic bacteria. There were no statistically significant upward or downward trends observed by Gram-positive aerobic or microaerophilic cocci or by Gram-negative aerobic bacilli. Similarly, no statistically significant trends were observed for multidrug-resistant bacteria [34]. In terms of diagnostic techniques, molecular assays are increasingly used in this field, improving our ability to detect rare pathogens, as in our case report [35,36]. We have learned from the field of periprosthetic joint infections that, especially in cases where microorganisms are exclusively cultured in sonication, isolating the causative microorganism is of utmost importance because an undetected infection and improper antibiotic treatment can lead to long-term prosthesis failure in the long run [37]. All available diagnostic methods should be considered in the field of septic arthritis to achieve the same goals.

### 3.4. Long-Term Treatment with Linezolid

Linezolid, a member of the oxazolidinone class of antibiotics, is commonly used to combat Gram-positive bacterial infections, including bacterial pneumonia, skin and soft tissue infections, and vancomycin-resistant Enterococcus faecium infections. Regarding the long-term safety of linezolid, it is generally well tolerated, with the most common adverse events being mild, reversible gastrointestinal upset, and skin reactions. Intolerance to linezolid occurs most commonly due to thrombocytopenia, gastrointestinal disturbances, decreases in hemoglobin/hematocrit, and skin reactions. The duration of therapy usually does not exceed 28 days. Data from the field of periprosthetic joint infections reported more prolonged treatment periods of up to 126 days. In terms of side effects associated with long-term treatment, the most common complications are hematological alterations (thrombocytopenia, anemia, and leukopenia) [38,39]. Soriano et al. [40] report data from eighty-five patients with an orthopedic implant infection treated with linezolid, with a median length of linezolid treatment in acute and chronic infections of 47 and 60 days, respectively. The most common adverse reactions were thrombocytopenia in four (4.7%) patients and anemia in five (5.8%) patients. When managing septic arthritis, it is essential to ensure that antibiotics can effectively penetrate the synovial fluid. In cases in which methicillin-resistant bacterial strains are suspected, linezolid may be used as a treatment option. With this in mind, researchers have explored the target site concentrations of linezolid in the synovial fluid to better understand its effectiveness better. According to a study by Schwameis et al. [41], linezolid was found to have good penetration into both knee gap and tissue, as evidenced by area under the ROC Curve (ACU) − AUCtissue/AUCfree plasma ratios of 0.76 ± 0.34 (synovial fluid) and 0.98 ± 0.62 (muscle tissue). Furthermore, in the synovial fluid, the AUC 0–24/MIC ratios for bacteria with an MIC of 1, 2, and 4 mg/L were 86.8 ± 47.0, 43.4 ± 23.5, and 21.7 ± 11.8, respectively. The authors concluded that intraarticular concentrations of linezolid might be optimal for the treatment of bacterial strains with an MIC90 of 1 mg/L. The mechanism of action of linezolid involves direct binding to the 50S ribosomal subunit, effectively inhibiting bacterial protein synthesis. This inhibition prevents the formation of a functional 70S initiation complex. While resistance to linezolid can develop due to slight modifications to the binding site on the ribosome, cross-resistance with other protein synthesis inhibitors is unlikely, given the drug’s unique mechanism of action. A recently published study by Vintila et al. [42] evaluated all adverse drug reactions to linezolid based on spontaneous reports from EudraVigilance (EV), the European database of suspected adverse drug reaction reports. The authors analyzed the ADR reports and found that by 31 December 2022, 13,381 adverse drug reactions had been reported for linezolid. This high number of reported ADRs may be attributed to various factors, including increased usage, extended treatment duration, and use in critically ill patients who are more susceptible to adverse reactions due to the presence of numerous risk factors. Additionally, the use of linezolid as an off-label indication, a frequent occurrence, might increase the risk of adverse drug reactions. In terms of *Clostridioides difficile* infection considered an adverse drug reaction, data from the same database, EudraVigilance, report only 66 cases [43]. Despite using linezolid for a prolonged period, we encountered no severe adverse drug reactions in our case, except for the increase in the liver enzymes for which the patient received hepatoprotective drugs, which normalized during the treatment period.

### 3.5. Limitations

Certain limitations in our case report should be considered, namely the lack of susceptibility testing of the isolated strains to penicillin or ampicillin in bacteriological studies and limitations that preclude the use of these cheaper and safer antibiotics in the management of our case.

## 4. Conclusions

Soil-borne bacterial pathogens such as *Bacillus pumilus* and *Paenibacillus barengoltzii* can pose a significant threat to human health. Utilizing matrix-assisted laser desorption–ionization time-of-flight mass spectrometry (MALDI-TOF MS) as a diagnostic technique can be an effective way to overcome these challenges and identify these organisms. International societies have recommended the use of molecular technology to aid in diagnosis, especially in the context of negative culture results. Our findings suggest that long-term treatment with linezolid, in combination with close monitoring of the patient, may be a viable option for the treatment of bone and joint infections caused by *Bacillus pumilus* and *Paenibacillus barengoltzii*. Additionally, cotrimoxazole and rifampicin can be used as oral alternatives.

## Figures and Tables

**Table 1:** Laboratory examinations performed at the time of admission.

Date	Parameter	Values	Reference Value
On admission	C-reactive protein	2.0 mg/L	0–0.3 mg/L
	Fibrinogen	382 mg/dL	180–350 mg/dL
	WBCs	9.93 × 10^3^/µL	4–9 × 10^3^/µL
	Differential blood count:		
	Neutrophils	6.86 × 10^3^/µL	1.5–7.5 × 10^3^/µL
	Lymphocytes	2.22 × 10^3^/µL	1.2–3.5 × 10^3^/µL
	Monocytes	0.59 × 10^3^/µL	0.2–0.8 × 10^3^/µL
	Basophils	0.03 × 10^3^/µL	0.01–0.15 × 10^3^/µL
	Eosinophils	0.12 × 10^3^/µL	0.02–0.06 × 10^3^/µL
	Red blood cells	5.56 × 10^6^/µL	4.6–6.2 × 10^6^/µL
	Hemoglobin	16.1 g/dL	14–18 g/dL
	Hematocrit	47.2%	40–52%
	Thrombocytes	304 × 10^3^/µL	150–450 × 10^3^/µL
	Coagulation tests	Prothrombin time (PT): 10.8 s	9.8–12.1 s
		Activated partial thromboplastin time (aPTT): 22.6 s	22.1–28.1 s
		International normalized ratio (INR): 1.04 s	0.86–1.2 s
	ESR	24 mm/h	0–15 mm/h
	Urea	28 mg/dL	15–39 mg/dL
	Creatinine	0.89 mg/dL	0.7–1.3 mg/dL
	Blood glucose	104 mg/dL	74–106 mg/dL
	Urine culture	Negative < 1000 CFU/mL	
	Alanine transaminase	38 U/L	16–63 U/L
	Aspartate transferase	117 U/L	15–37 U/L

**Table 2:** Antimicrobial susceptibility test results.

Antibiotic	MIC (μg/mL)	MIC Interpretation
***Bacillus pumilus***—beta-lactamases, ESBL-producing, carbapenemase, mannose-binding lectin, inducible resistance to clindamycin (negative)		
Ciprofloxacin	=0.5	Intermediate
Erythromycin	=1	Resistant
Levofloxacin	≤1	Intermediate
Linezolid	≤1	Sensitive
Meropenem	≤0.25	Sensitive
Vancomycin	≤1	Sensitive
***Paenibacillus barengoltzii***—beta-lactamases, ESBL-producing, carbapenemase, mannose-binding lectin, inducible resistance to clindamycin (negative)		
Ciprofloxacin	=0.5	Intermediate
Erythromycin	=0.5	Sensitive
Levofloxacin	≤1	Intermediate
Linezolid	≤1	Sensitive
Meropenem	≤0.5	Sensitive
Vancomycin	≤1	Sensitive

**Table 3:** Laboratory examinations performed during hospitalization.

Date	Parameter	Values	Reference Value
Day 1 after surgery	C-reactive protein	-	0–0.3 mg/L
	Fibrinogen	438 mg/dL	180–350 mg/dL
	WBCs	11.13 × 10^3^/µL	4–9 × 10^3^/µL
	Red blood cells	4.32 × 10^6^/µL	4.6–6.2 × 10^6^/µL
	Hemoglobin	12.5 g/dL	14–18 g/dL
	Hematocrit	36.5%	40–52%
	Thrombocytes	356 × 10^3^/µL	150–450 × 10^3^/µL
	ESR	-	0–15 mm/h
	Urea	22 mg/dL	15–39 mg/dL
	Creatinine	0.8 mg/dL	0.7–1.3 mg/dL
	Blood glucose	134 mg/dL	74–106 mg/dL
	Alanine transaminase	81 U/L	16–63 U/L
	Aspartate transferase	51 U/L	15–37 U/L
Day 3 after surgery	C-reactive protein	3.7 mg/L	0–0.3 mg/L
	Fibrinogen	586 mg/dL	180–350 mg/dL
	WBCs	11.09 × 10^3^/µL	4–9 × 10^3^/µL
	Red blood cells	4.61 × 10^6^/µL	4.6–6.2 × 10^6^/µL
	Hemoglobin	13.2 g/dL	14–18 g/dL
	Hematocrit	40.0%	40–52%
	Thrombocytes	342 × 10^3^/µL	150–450 × 10^3^/µL
	ESR	50 mm/h	0–15 mm/h
	Urea	37 mg/dL	15–39 mg/dL
	Creatinine	0.81 mg/dL	0.7–1.3 mg/dL
	Blood glucose	99 mg/dL	74–106 mg/dL
	Alanine transaminase	86 U/L	16–63 U/L
	Aspartate transferase	53 U/L	15–37 U/L
At the time of discharge	C-reactive protein	1.2 mg/L	0–0.3 mg/L
	Fibrinogen	367 mg/dL	180–350 mg/dL
	WBCs	6.1 × 10^3^/µL	4–9 × 10^3^/µL
	Red blood cells	4.78 × 10^6^/µL	4.6–6.2 × 10^6^/µL
	Hemoglobin	14.2 g/dL	14–18 g/dL
	Hematocrit	42.3%	40–52%
	Thrombocytes	342 × 10^3^/µL	150–450 × 10^3^/µL
	ESR	24 mm/h	0–15 mm/h
	Urea	33 mg/dL	15–39 mg/dL
	Creatinine	0.83 mg/dL	0.7–1.3 mg/dL
	Blood glucose	102 mg/dL	74–106 mg/dL
	Alanine transaminase	233 U/L	16–63 U/L
	Aspartate transferase	86 U/L	15–37 U/L

**Table 4:** Summary of the published cases of infections with *Bacillus pumilus*.

Author	Isolated Strain	Type of Infection	Antibiotic Susceptibility Test Results	Treatment
Fusini et al. [18]	*Bacillus pumilus*	C6–C7 intervertebral disc spondylodiscitis	Susceptible to ampicillin, amoxicillin, ciprofloxacin, clindamycin, erythromycin, imipenem, levofloxacin, teicoplanin, trimethoprim–sulfamethoxazole, and vancomycin	6 weeks IV amoxicillin/clavulanic acid 2.2 g q8H plus ciprofloxacin 400 mg q12H, then oral therapy for 6 weeks with amoxicillin/clavulanic acid 875/125 mg q8H plus ciprofloxacin 500 mg q12H
Borsa et al. [22]	*Bacillus pumilus*	Sepsis	Resistant to penicillin Susceptible to vancomycin, erythromycin, clindamycin, levofloxacin, and trimethoprim–sulfamethoxazole	IV ceftriaxone (1000 mg q12H) in association with metronidazole (500 mg q12H for 7 days followed by IV ceftriaxone (1000 mg q12H) for 14 days
Kimouli et al. [8]	*Bacillus pumilus* (*2 strains*)	Neonatal sepsis	Susceptible to penicillin, ampicillin, imipenem, vancomycin, erythromycin, levofloxacin, and trimethoprim–sulfamethoxazole and just one strain was susceptible to clindamycin	Vancomycin for 10 days

## Data Availability

All data generated or analyzed during this study are included in the published article.
